# Experience of Uncommon EGFR Mutation in Lung Scheme Quality Program: Discussing Risks and Opportunities for the Improvement of Laboratory Response

**DOI:** 10.1055/s-0041-1732481

**Published:** 2021-07-22

**Authors:** Alessandro Pancrazzi, Agostino Ognibene, Alice Moncada, Valerio Torre

**Affiliations:** 1 Department of Laboratory Medicine, Clinical and Molecular Pathology Unit, San Donato Hospital, Arezzo, Italy; 2 Department of Oncology, Pathological Anatomy Laboratory, San Donato Hospital, Arezzo, Italy

**Keywords:** uncommon EGFR mutations, TKIs, quality programs

## Abstract

**Background** The quality programs can be considered to be a valuable tool for global and individual growth. Each result, obtained by a single laboratory, contributes to define the standardization of the response. In the case of the uncommon epidermal growth factor receptor (EGFR) mutations, the molecular result is sometimes difficult to interpret in terms of biological significance and therapy choosing. The standardization effort in the diagnostic lung setting also consists of active quality program participation.

**Materials and Methods** The quality control analysis, which is defined as a clinical case, was performed by the extraction of DNA from FFPE sections and by RT-PCR on the EGFR (exons 19, 20, 21), BRAF, and KRAS genes. The laboratory performed a validation sequencing of EGFR exon 20 with the help of the Sanger method.

**Results** The laboratory reported positivity for EGFR exon 20 insertions and negative results for BRAF and KRAS. The quality test finished with the redaction of a report containing the recommendation to consider the efficacy of therapy with tyrosine kinase inhibitors (TKI). This specific interpretation has determined poor performance judgment by the quality provider, which explained why most of these mutations are TKI-resistant.

**Conclusions** This experience provides an opportunity to reflect on the critical aspects of this diagnostic setting. The detection of some uncommon EGFR mutations should entail the mutation characterization, especially for the rare exon 20 insertions, of which are not classifiable as “resistant.” Moreover, this experience allows reflecting on the quality program design, mandatory actions for the laboratory, and routine activity in the oncologic multidisciplinary team.

## Introduction

In the target therapy era, every single molecular pathology laboratory must take note of the newly available therapeutic strategies to provide a useful clinical report for correct clinical management. This concept is particularly important in lung staging. Epidermal growth factor receptor (EGFR) gene test should distinguish sensitive and resistant mutations to choose correct therapeutic approaches. The European Molecular Genetics Quality Network (EMQN) initiative represents a valid point to assess laboratory performance and check individual center skills in the global oncology setting. Following the good laboratory practice for the quality check activity, each result and performance must be discussed to identify critical or strong points of the team and increase the appropriateness of the response. The “poor” quality of a single result, like the one obtained from the local laboratory in the last round of the EMQN Lung exercise, has imposed a deep internal survey that pushed to evaluate the meaning of uncommon and sensitive EGFR mutations. The laboratory should solve the single critical technical aspects, but this is not a unique challenge. This quality round highlighted points like the term choosing for clinical reports and the sequential steps of the setting's procedure. These last points represent a determining side of the global analysis. In our case, the critical aspect which emerged from EMQN quality check has been the interpretation comment in the clinical report. Uncommon EGFR mutations, like ins 20, are mostly resistant to the tyrosine kinase inhibitors (TKI), but genotyping of a single variant must be evaluated to provide specific and pertinent indications.

## Materials and Methods

The analysis sample consisted of one section of formalin-fixed paraffin-embedded (FFPE) tumor with > 20% neoplastic cells. Quality scheme documents reported the following reason for request/clinical indication: This female patient showed, in the positron emission tomography (PET)-CT scan, a metabolically active mass in the Segmentumapicoposterius left. Endobronchial ultrasound-guided transbronchial needle aspiration was performed, and the pathology assessment revealed malignant cells of a TTF-1 positive low differentiated adenocarcinoma. The material was assessed as suitable for molecular analysis. The tissue extraction was performed by Promega FFPE DNA kit (AS1450), and the DNA quantity was measured by Nanodrop Instrument. Extracted DNA amounted to 275 ng/microliter. The laboratory performed mandatory test for EGFR status and facultative examinations for KRAS and BRAF genes. The polymerase chain reaction (PCR) methods were executed by EasyPGX system (Diatech Pharmacogenetics, Jesi, Italy). This assay allows detecting EGFR exon 20 insertions but does not distinguish among these. The exon 20 EGFR validation method, performed postsubmission of results to EMQN, underwent bidirectional Sanger sequencing by SeqStudio instrument (Thermo Fisher Scientific, MA, USA). The oligonucleotides used for the Sanger method responded to homemade design.

The molecular report, which was submitted to the quality provider, consisted of several parts. The first section contains personal details, sample tracking data, and other information (forename, surname, date of birth, sex, laboratory ID reference, EMQN ID, sample type, reason for request/clinical indication, test (s) requested, reception date, sending and result microscopy). The second section reports molecular results and examined targets. The final part of the document contains interpretation and method/limits. The molecular report ends with performers' identity, their signature, and date of document production.

## Results

The EGFR investigation allowed identification of wild type status for 18, 21 and 19 exons and detection of mutation on exon 20. The sample resulted positive for exon 20 insertion. No BRAF and KRAS mutations were detected.

The validation of reverse transcriptase polymerase chain reaction (RT-PCR) EGFR positive results was performed after EMQN submission through Sanger sequencing and allowed to identify the following variant: c.2305_2308delinsCTGGACAACCCCC p. Val769_Asp770delinsLeuAspAsnProHis). This result did match up with the global outcome of the quality program.

### Reporting and Interpretation

The laboratory clinical report was submitted to the quality provider. This document reported the positivity for EGFR exon 20 insertion without specifying the mutation type due to limits of the RT-PCR assay.

The section on interpretation included the following: Molecular analysis of the EGFR gene allowed to detect exon 20 insertion pathogenetic variant. BRAF and KRAS tests resulted in negative, wild-type status. The mutant status of EGFR allows to consider the efficacy of therapy with TKI and possibly monitor EGFR status to prevent resistance evolution due to possible future mutation EGFR p. Thr790Met.

### Quality Scheme Result on the Sample and Communications between Laboratory and Provider

Forty-two days after submission of results, the quality provider published a summary scheme report (preappeals) and allowed to submit an appeal into a temporal range of 27 days. The summary scheme report, naturally, did not contain the individual performance of the participants but reported generical errors related to several criteria of response: genotyping, interpretation, and clerical accuracy.

The laboratory staff read this document and took note that the global performance was affected by four types of error in the “comment with deduction” section. The quality provider assigned these mistakes four gravity levels (deducted score: 0.2–0.5–1.0–1.5) but did not explain specific reasons linked to the different scores. While the participant laboratory was not able to download the appeal form, because of the browser configuration, an appeal was not finally submitted by choice, considering the clinical report was rightly redacted.

Two weeks from the expiration date, the laboratory received the score of the performance. The participant laboratory learned from this documentation that its own score for lung scheme was considered “poor” for mistakes in the interpretation section. The laboratory, in reply to the explanation requested, received clarification. The critical point determining this poor result was as follows: critical interpretation error because exon 20 insertion tumors are mostly resistant to TKI (deducted points 2).

The laboratory manager answered this contest by explaining two main points: not all uncommon EGFR exon 20 insertions should be considered resistant to TKI, and the clinical report is not an exhaustive instrument for patient staging, as each case is usually discussed in a multidisciplinary team.

Moreover, the laboratory manager answered that the comment phrase on the potential use of TKI, reported in the laboratory report, should be related to the generic result of exon 20 insertion. The following evidence should be considered in case of these uncommon mutations:

- Pasi et al. Antitumor activity of TAK-788 in NSCLC with EGFR exon 20 insertions. Journal of Clinical Oncology 2019 37:15_suppl, 9007–9007
- Hirano et al. In vitro modeling to determine mutation specificity of EGFR tyrosine kinase inhibitors against clinically relevant EGFR mutants in non-small-cell lung cancer. Oncotarget. 2015 Nov 17;6(36):38789–803.
- Lin Y-T and Shih J-Y. Not all EGFR exon 20 insertions are created equal. JTO Clin Res Rep 1:100069.

The reply finally considered that the local clinical setting attempts to update itself on treatments, and in these specific cases, it is important to try to overcome the idea of uncommon mutations and consequently complete exclusion of all TKI.

The quality program staff accurately evaluated this reply and commented that the laboratory manager may probably have a point, but the appeal time was expired, so the laboratory score for the lung scheme remained poor.

### Corrective/Preventive Actions by the Laboratory

The laboratory discussed this result and analyzed all the possibly linked critical aspects. As a result, the following main corrective actions were done:

- Interpretation comments will be reported by the laboratory manager and checked by another colleague to verify both concordance with the result and message clarity.
- Every data emission and reciprocal communication for quality schemes will be managed in double-check system by two colleagues to reduce misunderstanding risks, typos, or technical problems due to digital platform.
- The laboratory will develop next-generation sequencing (NGS) tests for routine detection of the oncologic markers.
- The laboratory manager and his attendant will participate even more closely in the oncological multidisciplinary team to discuss the interpretation of the molecular results and follow the final decision in clinical management.
- The potential clinical risk of this event was discussed among the participants of the multidisciplinary oncology team.

## Conclusions and Prospects

Most of the scientific literature about EGFR exon 20 insertions has reported insensitivity to EGFR-targeted drugs. The TKI therapy experience generally highlights that the progression-free survival in these cases is drastically lower than the one with common EGFR mutations. 1 2 3 4 However, several results of the treatment response and data of pharmacological effects showed promising evidence to consider TKI as one of the therapy options for some ins20 EGFR mutations. 5 6 7 8 9 10 Moreover, different clinical trial data of new generation TKI suggest to take into account an effective response in these mutation types. The significant efficacy of the TKI for EGFR uncommon exon 20 mutations is reported for the following molecules: poziotinib, mobocertinib (TAK-788), amivantamab (JNJ-61186372, JNJ-6372), DZD9008 and TAS6417/CLN-08110. 11 12 13 14 15 The therapeutic approach for some ins20 mutations also involves pharmacological combinations of immunotherapy and TKI (e.g., cetuximab). This therapeutic scenario, even if it does not place TKI as the main therapeutic choice, does not exclude all the available generation TKI.

The potential application of TKI and uncommon EGFR provides exact genotyping of the genetic alteration. One of the most useful methods to detect EGFR mutations is the multiplex RT-PCR assay, which is a fast, sensitive, and efficient method to screen positive patients. 16 The sensitivity of this methodology allows conducting a deep investigation, really helpful in lung samples from small biopsies or cytology frequently affected by poor cellular representativeness. Unfortunately, this test typology is not able to distinguish exon 20 single variants and consequent sequencing is necessary to determine the exact nature of the alteration. In the specific case of the EGFR exon 20 insertions, the screening positivity leads to several therapeutic options, among which the most appropriate finally rely on knowledge of the exact mutation. The turnaround time of the laboratory is a crucial aspect due to the clinical necessity to urgently design a therapeutic plan for the patient. In the case of EGFR negativity, the multiplex RT-PCR provides a fast valid response, but in the case of positivity event, the complete laboratory response time is linked to a second level methodology such as the Sanger sequencing. Currently, the use NGS technology is a valid solution to not reduce exhaustive laboratory responses but decrease response time. The widespread NGS oncologic panel allows the laboratory to rapidly provide the nature of the target mutation of the EGFR gene. Considering the necessity to cover the genic regions and provide the genotype of each mutation, this approach should be considered mandatory for the most routine analyses. The spreading limits of this technology, due to the costs and to the specialized laboratory staff, have been overcome today. There is a wide availability of commercial kits whose costs, considering coverage needs, are more affordable compared to the conventional methods. 17 Moreover, the professional formation of the laboratory staff operating in the clinical molecular pathology sector has grown over several years, with the geneticist being usually involved.

The critical point of this experience is mainly represented by the clinical report style. In truth, this emerging point is connected to the specific relationship between the laboratory and clinical sector. The real problem has been the lack of universal clarity of the given report. Even if, in this specific case, there is minimal clinical risk, because the real process is usually concluded with an accurate final clinical report, which is redacted in agreement among various specialists, nevertheless it is not completely acceptable to release an official “general interpretation,” not even into a quality simulation. The local multidisciplinary team concluded that the critical event which occurred should not be considered a serious risk for patient management, because the oncologists always read the entire clinical report and consider genotyping results in its entirely (in this case, positively evaluated by the quality provider). Moreover, the oncologist has the responsibility to provide clarifications about interpretation within a periodic and punctual oncologic multidisciplinary team, personalized for each patient, in which the pathologist talks about every single result and its clinical significance. In addition to these considerations, we must consider that a clinical report is not the unique step to provide for therapy assessment, since the modern center is organized in a multidisciplinary team, in which each case is discussed with the pathologist. Regardless of the mutational genotype, the global clinical status, the clinical safety, and the costs/benefits ratio for a specific therapy must be considered.

One of the final aims of a quality program must naturally be the process check, and in this specific case, the laboratory has been ready to discuss and face the critically emerging points. After all, it seems necessary to think about the educational aspect of several quality programs. The scientific guideline, quality program, and diagnostic kit manufacturer, all recommend identifying a single positive result among several possible ones in the large region examined (like EGFR exon 20). The reason for this recommendation is clear because, over several years, the target therapy research increased the available drugs list, and the new clinical trials have provided several possible alternatives to the chemotherapy approach, depending on the mutation nature. In past decades, the major problem lay in detecting the alteration status. 18 At this point in time, the need to report the mutation nature should be strongly highlighted. The minimally acceptable analytical result in this interlaboratory program has been general genotyping; in this experience, the participant laboratory passed the genotyping test, but should probably demand the specific mutation nature. The approved RT-PCR methods, based on multiplex design, involve the acceptance of the generic detection without mutation type characterization. In this evaluation setting, it is not always possible to provide a conclusive and exhaustive interpretation. A quality test should be able to measure the entire activity of the center, but is not properly possible to evaluate clinical management, based exclusively on these premises. This individual EMQN experience suggests thinking about these critical points. In the future, considering the close relationship among various phases of the process (preanalytical, genotyping, and interpretation), it is desirable to increase the educational aspect in relation to each analytical quality test. In the case of exon 20 EGFR mutations, the sequencing approach is particularly pertinent and seems to be a unique way to completely observe scientific guidelines and recommendations and impose exhaustive laboratory responses. Taking into account that the exon 20 insertions are approximately 40% of rare EGFR mutations, we always should consider the sequencing of all these uncommon ones. 19 This action should be strongly recommended and added to minimal criteria of the quality program schemes to make sure each laboratory provides the sequence information necessary for the personalized medicine setting.

Finally, we should encourage active participation in quality programs. This laboratory experience highlights the relevance of the quality program participations such as EMQN. These occasions are not only important to assess analytical performance but also evaluate all setting processes and analyze aspects that could potentially affect global clinical management. Even if guidelines contain several recommendations, it is often difficult to analyze secondary aspects of the entire workflow. An efficient quality program allows, according to the quality system principles, the participant to convert a potential procedure risk into an improvement opportunity.

We would like to acknowledge the technical support of the following staff of the San Donato Hospital, Arezzo, Italy: Drs. Perticucci Roberta, Vecchietti Stefania, and Lenzi Silvia of the clinical molecular pathology sector of the analysis laboratory.

## References

[1] Wu J Y Wu S G Yang C H Lung cancer with epidermal growth factor receptor exon 20 mutations is associated with poor gefitinib treatment response Clin Cancer Res 2008 14 15 4877 4882 18676761 10.1158/1078-0432.CCR-07-5123

[2] Greulich H Chen T H Feng W Oncogenic transformation by inhibitor-sensitive and -resistant EGFR mutants PLoS Med 2005 2 11 e313 16187797 10.1371/journal.pmed.0020313 PMC1240052

[3] Oxnard G R Lo P C Nishino M Natural history and molecular characteristics of lung cancers harboring EGFR exon 20 insertions J Thorac Oncol 2013 8 02 179 184 23328547 10.1097/JTO.0b013e3182779d18 PMC3549533

[4] Zhang T Wan B Zhao Y Treatment of uncommon *EGFR* mutations in non-small cell lung cancer: new evidence and treatment Transl Lung Cancer Res 2019 8 03 302 316 31367543 10.21037/tlcr.2019.04.12 PMC6626855

[5] Hirano T Yasuda H Tani T In vitro modeling to determine mutation specificity of EGFR tyrosine kinase inhibitors against clinically relevant EGFR mutants in non-small-cell lung cancer Oncotarget 2015 6 36 38789 38803 26515464 10.18632/oncotarget.5887 PMC4770737

[6] Jia Y Juarez J Li J egf816 exerts anticancer effects in non-small cell lung cancer by irreversibly and selectively targeting primary and acquired activating mutations in the EGF receptor Cancer Res 2016 76 06 1591 1602 26825170 10.1158/0008-5472.CAN-15-2581

[7] Yasuda H Park E Yun C H Structural, biochemical, and clinical characterization of epidermal growth factor receptor (EGFR) exon 20 insertion mutations in lung cancer Sci Transl Med 2013 5 216 216ra177 10.1126/scitranslmed.3007205 PMC3954775 24353160

[8] Voon P J Tsui D W Rosenfeld N Chin T M EGFR exon 20 insertion A763-Y764insFQEA and response to erlotinib--Letter Mol Cancer Ther 2013 12 11 2614 2615 23969006 10.1158/1535-7163.MCT-13-0192

[9] Woo H S Ahn H K Lee H Y Epidermal growth factor receptor (EGFR) exon 20 mutations in non-small-cell lung cancer and resistance to EGFR-tyrosine kinase inhibitors Invest New Drugs 2014 32 06 1311 1315 25146938 10.1007/s10637-014-0146-x

[10] Yang J C Sequist L V Geater S L Clinical activity of afatinib in patients with advanced non-small-cell lung cancer harbouring uncommon EGFR mutations: a combined post-hoc analysis of LUX-Lung 2, LUX-Lung 3, and LUX-Lung 6 Lancet Oncol 2015 16 07 830 838 26051236 10.1016/S1470-2045(15)00026-1

[11] Robichaux J P Elamin Y Y Tan Z Mechanisms and clinical activity of an EGFR and HER2 exon 20-selective kinase inhibitor in non-small cell lung cancer Nat Med 2018 24 05 638 646 29686424 10.1038/s41591-018-0007-9 PMC5964608

[12] Riely G J Neal J W Camidge D R Activity and safety of mobocertinib (TAK-788) in previously treated non-small cell lung cancer with EGFR Exon 20 insertion mutations from a phase 1/2 trial Cancer Discov 2021 11 07 1688 1699 33632775 10.1158/2159-8290.CD-20-1598 PMC8295177

[13] Yun J Lee S H Kim S Y Antitumor activity of amivantamab (JNJ-61186372), an EGFR-MET bispecific antibody, in diverse models of *EGFR* Exon 20 insertion-driven NSCLC Cancer Discov 2020 10 08 1194 1209 32414908 10.1158/2159-8290.CD-20-0116

[14] Yan X Lin Z Lifang Z Yingchun W Mei W Zhenfan Y DZD9008, an oral, wild type selective EGFR inhibitor for the treatment of non-small-cell lung cancer with Exon20 insertion and other activating mutations Cancer Res 2019 10.1158/1538-7445.AM2019-3081

[15] Udagawa H Hasako S Ohashi A TAS6417/CLN-081 is a pan-mutation-selective EGFR tyrosine kinase inhibitor with a broad spectrum of preclinical activity against clinically relevant *EGFR* mutations Mol Cancer Res 2019 17 11 2233 2243 31467113 10.1158/1541-7786.MCR-19-0419 PMC6872223

[16] Ellison G Zhu G Moulis A Dearden S Speake G McCormack R EGFR mutation testing in lung cancer: a review of available methods and their use for analysis of tumour tissue and cytology samples J Clin Pathol 2013 66 02 79 89 23172555 10.1136/jclinpath-2012-201194 PMC3582044

[17] Beau-Faller M Prim N Ruppert A M When should we order a next generation sequencing test in a patient with cancer? EClinicalMedicine 2020 25 100487 32775973 10.1016/j.eclinm.2020.100487 PMC7397394

[18] Patton S Normanno N Blackhall F Assessing standardization of molecular testing for non-small-cell lung cancer: results of a worldwide external quality assessment (EQA) scheme for EGFR mutation testing Br J Cancer 2014 111 02 413 420 24983368 10.1038/bjc.2014.353 PMC4102953

[19] Beau-Faller M Prim N Ruppert A M Rare EGFR exon 18 and exon 20 mutations in non-small-cell lung cancer on 10 117 patients: a multicentre observational study by the French ERMETIC-IFCT network Ann Oncol 2014 25 01 126 131 24285021 10.1093/annonc/mdt418 PMC3868323

